# A Novel p. Gly630Ser Mutation of *COL2A1* in a Chinese Family with Presentations of Legg–Calvé–Perthes Disease or Avascular Necrosis of the Femoral Head

**DOI:** 10.1371/journal.pone.0100505

**Published:** 2014-06-20

**Authors:** Na Li, Jian Yu, Xiang Cao, Qiu-Yue Wu, Wei-Wei Li, Tian-Fu Li, Cui Zhang, Ying-Xia Cui, Xiao-Jun Li, Zhi-Min Yin, Xin-Yi Xia

**Affiliations:** 1 Institute of Laboratory Medicine, Jinling Hospital, Nanjing University School of Medicine, Nanjing, PR China; 2 Department of Orthopaedics, Jinling Hospital, Nanjing University School of Medicine, Nanjing, P.R. China; The University of Hong Kong, Hong Kong

## Abstract

**Objective:**

Mutations in the type II collagen gene are associated with certain human disorders, collectively termed type II collagenopathies. They include Legg–Calvé–Perthes disease (LCPD) and avascular necrosis of the femoral head (ANFH). These two diseases are skeletal dysplasias, inherited in an autosomal dominant fashion, characterized by groin pain, dislocation of the hip and diminished joint mobility. Coxa vara and elevation of the greater trochanter of the femur comprise the typical phenotype of LCPD, but do not occur in ANFH. Lack of synthesis of type II collagen and structural defects are responsible for the major clinical outcomes, because collagen is the essential matrix protein of all connective tissues. Type II collagen, encoded by the *COL2A1* gene, contains N- and C- terminal regions that are cleaved after secretion into the extracellular matrix, and the core area is composed of a triple helical (Gly–X–Y) domain. If the Gly in this specific region is replaced by other amino acids, the structure of type II collagen will be destroyed.

**Method:**

Forty-five members of a four-generation family were recruited and investigated. Diagnosis was made by independent orthopedic surgeons and radiologists. A mutation of the *COL2A1* gene was detected.

**Result:**

In our research, we identify a heterozygous mutation (c.1888 G>A, p. Gly630Ser) in exon 29 of *COL2A1* in the Gly–X–Y domain, in a Chinese family affected by LCPD and ANFH. Our findings provide significant clues to the phenotype–genotype relationships in these syndromes and may be helpful in clinical diagnosis. Furthermore, these results should assist further studies of the mechanisms underlying collagen diseases.

**Conclusion:**

Our data add new variants to the repertoire of *COL2A1* mutation resulting in related collagenopathies.

## Introduction

Recent genetic studies have demonstrated that mutations in the type II collagen gene (*COL2A1*) may result in certain human disorders collectively termed type II collagenopathies. These conditions are expressed as a continuous spectrum of phenotypes, ranging from perinatally lethal mutations, to severe disease, to cases with only mild arthropathy. Type II collagen is the prominent collagen found in articular cartilage [1], [2]. Articular cartilage provides a nearly frictionless surface and acts as a load absorber during movement [3]. There are many reported phenotypes of type II collagenopathies, avascular necrosis of femoral head (ANFH OMIM#608805) [4], spondyloperipheral dysplasia congenita (SEDC #183900) [5], and Legg–Calvé–Perthes disease (LCPD OMIM#150600) [6], [7]. The above disorders have significant similarities in phenotypic and radiologic traits. ANFH is a consequence of impaired blood supply [8], which is the most typical type II collagenopathy resulting from mutations of the type II collagen gene (*COL2A1* OMIM#120140). The clinical manifestations of ANFH, including pain on exertion, a limping gait, and a discrepancy in leg length, cause considerable disability [9]. ANFH generates a variety of disorders, most commonly trauma, development dysplasia of the hip, slipped capital femoral epiphysis etc. It has been reported that bilateral degenerative hip disease in children is commonly recognized in cases of LCPD, the peak incidence of which occurs between the ages of 4 and 8 years [10]. This is a type of avascular necrosis of the femoral head that causes early osteoarthritis. The typical characteristics of LCPD are coxa palan (coxa vara and elevation of the greater trochanter of the femur) [10]. Almost all *COL2A1* gene-related diseases result from a single collagenopathiy. Although some correlations have been determined between the location of the mutation and disease phenotype, the genotype-phenotype correlations in type II collagenopathies are still far from clear.

The *COL2A1* gene provides a key element for the production of type II collagen, which is found in cartilage, bone, the nucleus pulposus, and the vitreous of the eyes [11], [12], and is localized at 12q13.11-q13.2 [13]. The type II collagen gene is about 30 kb in length with 54 exons, among which exons 1–5 encoding the N- terminal region, exons 6–48 encode the core area which is composed of a triple helical domain, and exons 49–52 encoding the C- terminal region. Three α1 chains encoded by the *COL2A1* gene are folded together in a triple-helical configuration to form the procollagen homotrimer. The N- and C- terminal amino acid sequences called propeptides are cleaved after the secretion of procollagen in the extracellular matrix [14]. The triple helical domain, containing about 300 amino acids, exists in the limited space of the procollagen, and is restricted so that every three amino acids is a glycine, generating a repeating (Gly–X–Y)_n_ sequence pattern. If the Gly in this specific region is replaced by other amino acids, the structure of type II collagen will be destroyed. Over 200 different mutations have been found in the *COL2A1* gene; the most common type is the substitution of the triple-helical glycine residues. Until now, no mutation hotpots have been confirmed [15]. Although many studies have shown that significant mutations exist in the *COL2A1* gene, the identification of gene mutations in Chinese family members with ANFH or LCPD is rare. It is therefore important to determine the gene mutations in Chinese patients with these diseases and therefore contribute to clinical diagnosis and treatment. The purpose of our study is to identify the genetic abnormality responsible for ANFH and LCPD in a single family and to determine the factors responsible for the distinct phenotypes manifested by different family members.

## Materials and Methods

### 2.1 Ethics Statement

The Ethics Committee of Jinling Hospital approved the protocols used. The individuals described in this manuscript have given written informed consent (as outlined in PLOS One consent form) to publish these case details.

### 2.2. Case Presentation and Analysis

**Figure 1:**
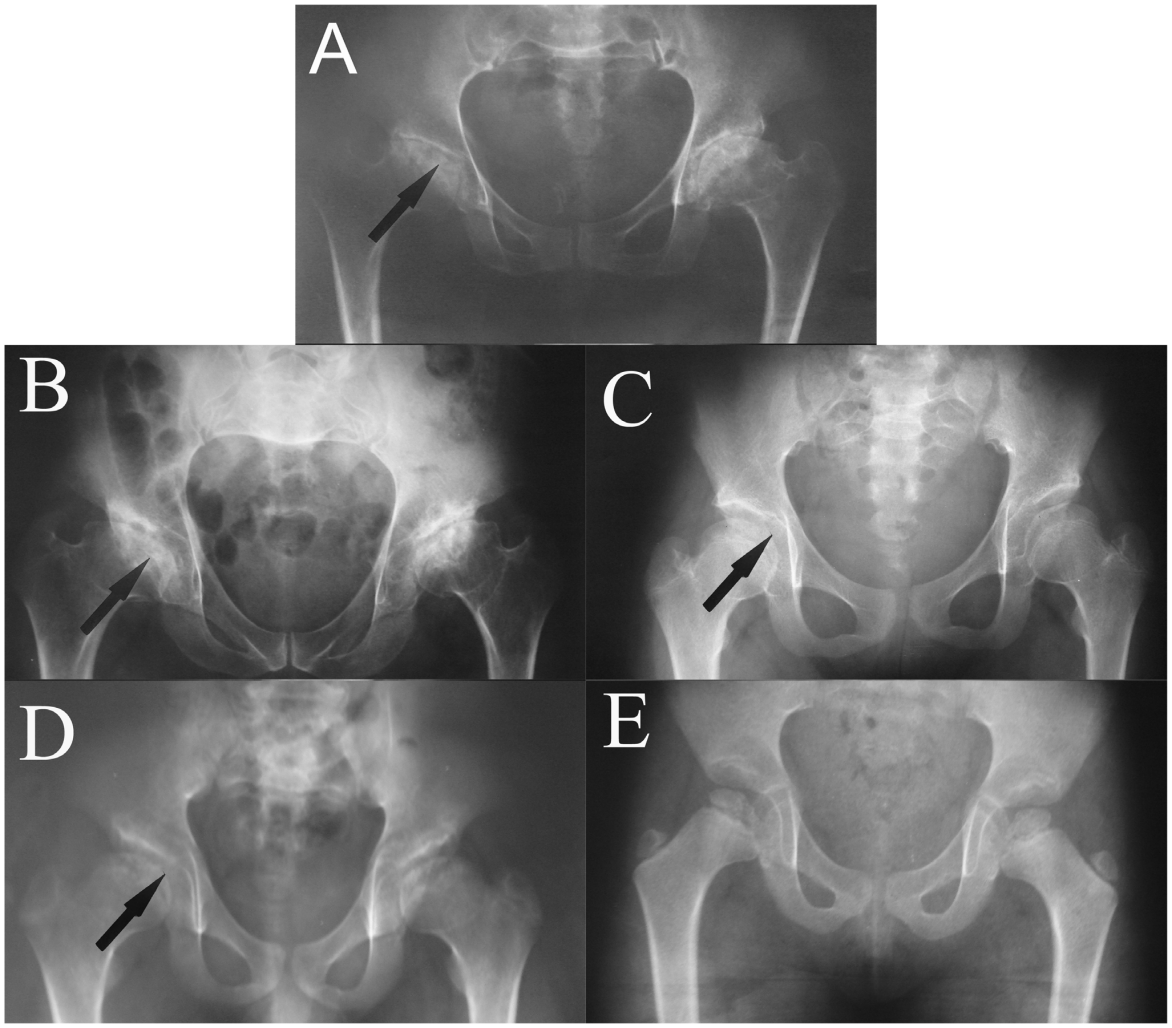
Radiograph of the family members. Radiographs of the pelvis of the family members III-6 (A) and III-7 (B), diagnosed with LCPD, reveals abnormalities of the capital femoral epiphyses, flattening of the acetabular roof with cystic degeneration, coxa plana (coxa vara and elevation of the greater trochanter of the femur) (**arrow**). The radiographs of the hip in family member IV-7 (C) and IV-8 (D) shows partial collapse of the femoral head, without coxa plana (**arrow**); the patient was diagnosed with ANFH. The radiograph of the family member IV-9 (E) did not exist typical manifestations. In addition, The X-ray of the family members III-6, III-7, IV-7, IV-8, IV-9 were made at the age of 41, 38, 15, 15 and 7 years old respectively.

The proband (III-6), a 41-year-old woman with LCPD accompanied by osteoporosis (OP), came to our hospital for genetic counseling with regard to bone disease. Her condition was characterized by groin pain, a short femoral neck and flattened epiphyses. Detailed records of her medical history, and physical and laboratory examinations had been obtained. The major pathological changes present in the hip joint were coxa plana, flat femoral head with cystic degeneration and secondary OP (Fig. 1A). The X-ray of her spine was normal and her facial features were unremarkable. Neurological examinations revealed no significant findings and there were no obvious abnormalities in her limbs. The family member III-7 had the same manifestations (Fig. 1B).

**Figure 2:**
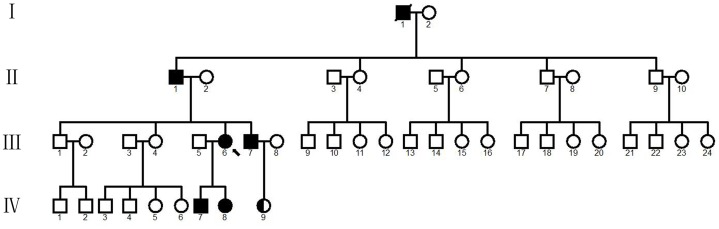
The pedigree of a Chinese family with LCPD and ANFH. The arrow indicates the proband (III-6). The white boxes represent healthy males and white circles represent healthy females. Black boxes represent affected males and black circles represent affected females. The box with half white and half black indicate the status of carrier, including an asymptomatic 7-year old girl (IV-9). The box with a crossing line indicated that the person has already died. The diagnoses of LCPD was made in members III-6 and III-7; of ANFH in members IV-7 and IV-8.

The second patient (IV-7), a 15-year-old boy, first reported groin pain and a limp at age 6 years, whose clinical diagnosis was ANFH. ANFH and LCPD were diagnosed using internationally recognized criteria. Examination showed restricted movement of the hips bilaterally; radiography confirmed femoral head collapse and subluxation. There was no coxa plana (Fig. 1C). The patient IV-8 contained the same phenotype (Fig. 1D). There was not typical abnormal in IV-9 (Fig. 1E) The pedigree of the family is shown in Fig. 2, a diagnosis of LCPD was made in member III-6 and III-7, and of ANFH in members IV-7 and IV-8 (Table 1). The family member I-1 was already dead, and II-1, almost 80 years old, was paralyzed accompanied by significant hip pain, so we could not get the related radiograph materials. No obvious abnormalities were seen in the limbs of the affected subjects in this family. The control group consisted of 200 healthy volunteers showed no abnormalities on physical, neurological and radiological examinations.

**Table 1:** Phenotype details of the Chinese family with LCPD or ANFH.

Family member/age/sex/height(cm)	Age at onset,years	Age at diagnosis,years	Majorsymtoms	Radiographic findings	Phenotypegroup
III-6/40/F/148	5	40	Limping/groin pain	Cystic/collapse/subluxation	LCPD/OP
III-7/38/M/158	6	38	Limping/groin pain	Cystic/collapse/subluxation	LCPD/OP
IV-7/15/M/143	10	15	Limping/groin pain	Marginal osteophytes/joint spacenarrowed	ANFH
IV-8/18/F/139	8	15	Limping/groin pain	Cystic	ANFH
IV-9/7/F/−	–	7(asymptomaticcarrier)	Normal	Normal	–

### 2.3. *COL2A1* Gene Mutation Analysis

Informed consent was obtained from the family members and 200 healthy volunteers before DNA analysis. All protocols were carried out by the Institutional Review Board and the Committee on Ethics of Research Involving Human Subjects of Nanjing University Medical Center, Nanjing, China. A Wizard™ Genomic DNA Purification Kit (Promega, Madison, USA) was used to extract leucocyte genomic DNA, according to the manufacturer’s instructions and all samples were stored at –20°C. The primers were designed using Primer 5 software based on the sequences of 54 exons and the exon–intron boundaries of the *COL2A1* gene. Polymerase chain reaction (PCR) was performed under the following conditions: 95°C for 5 min followed by 35 cycles of 94°C for 30 s, 56°C for 30 s, and 72°C for 60 s; the products were then sequenced by BGI. The sequencing results were compared to the NCBI Reference Sequence (NG_008072.1). Protein sequence alignments with partial type II collagen sequences from multiple species (*Homo sapiens, Mus musculus, Rattus norvegicus, Canis lupus familiaris, Oryctolagus cuniculus and Gallus gallus*) were analyzed by DNAMAN software.

## Results

**Figure 3:**
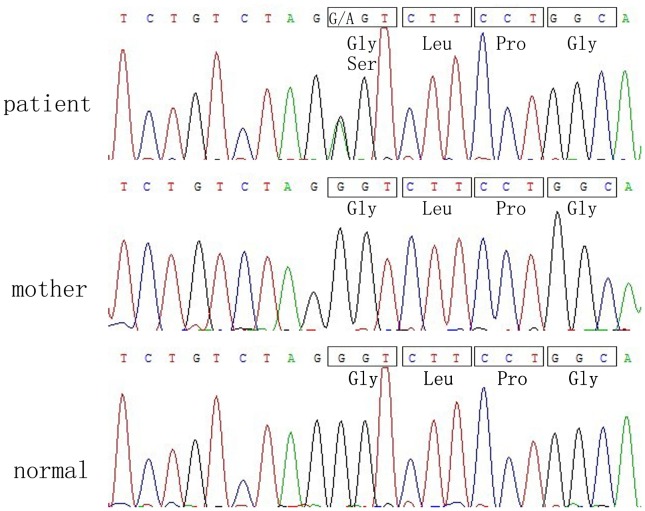
Analysis of sequences of the partial *COL2A1* gene. Novel mutation c.1888G>A in exon 29 of *COL2A1* was identified in the proband, which may have resulted in a substitution of glycine to serine at codon 860 (p.Gly630Ser); the arrow indicates the heterozygous mutation (c.1888G>A). It was discovered that the proband and affected members of the family possess the same mutation site (c.1888G>A), however other members of the family and the 200 normal controls did not.

We discovered that the proband with LCPD accompanied by OP carried a novel substitution mutation, which consisted of a heterozygous G-to-A transition (c. 1888 G>A) (Fig. 3) in exon 29 of the *COL2A1* gene. This may result in a glycine-to-serine substitution at position 630 (p. Gly630Ser) of type II collagen. The six affected family members were also analyzed for mutations in *COL2A1*, and the same heterozygous *COL2A1*gene mutation (c. 1888 G>A) was identified. This same mutation site was not found in the DNA samples from the healthy family members and volunteers, and other sequence changes were not observed.

**Figure 4:**
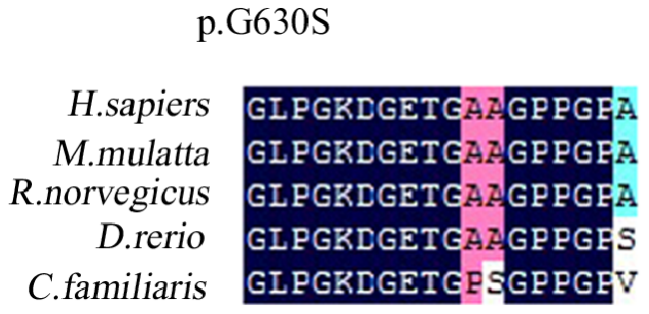
Protein sequence alignments with multiple species. The novel mutation (c.1888 G>A) in exon 29 of *COL2A1* may resulti in changes in the protein (p.G630S) is highly conserved across species, including *H.sapiens, M.mulatta, R.norvegicus, D.rerio,* and *C. familiaris.* Multispecies alignment of *COL2A1* orthologs shows that the mutation site (indicated with an arrow) identified in the proband alters a highly conserved amino acid.

The Gly630 in the triple helical domain of type II collagen, encoded by the *COL2A1* gene, was highly conserved among species including *Homo sapiens, Mus musculus, Rattus norvegicus, Canis lupus familiaris, Oryctolagus cuniculus and Gallus gallus* (Fig. 4).

## Discussion

We identified a glycine substitution mutation (c. 1888 G>A) of *COL2A1* resulting in LCPD or ANFH in a Chinese family, which had not been reported previously. This mutation exists in affected individuals and its absence from unaffected family members and controls demonstrates its causative role in the disease phenotype seen. LCPD is a group of rare inherited chondrodysplastic disorders which is a common hip disorder in children characterized by coxa plana (coxa vara and elevation of the greater trochanter of the femur), dislocation of the hip and diminished joint mobility. It differs from the manifestation of ANFH, which does not include coxa plana. It was reported there were four distinctive stage growth disturbance, subchondral fracture and fragmentation, re-ossification and healed or residual which formed a cycle, thus LCPD is a self-limiting and self-healing disorder, while ANFH are different. The occurrence of *COL2A1* gene mutations in Chinese patients with two different diseases related to type II collagen is rare. The major mutation sites of *COL2A1* have been found to be glycine substitutions in the triple helical domain of type II collagen [16], [17]. The triple-helical domain of type II collagen is characterized by about 330 repeating Gly–X–Y triplets. Researches have found that the Gly630 in this region is highly conserved among species including *Homo sapiens, Mus musculus, Rattus norvegicus, Canis lupus familiaris, Oryctolagus cuniculus and Gallus gallus*. Although the major mutations involve replacement of glycine in the Gly–X–Y triple helical domain, the severity of the clinical manifestations varies greatly. The serine-to-glycine substitution fails to result in lethal phenotype, probably because the two amino acids are polar. However the mutation adds a large hydroxymethyl group to the center of the superhelix, which is predicted to disrupt the local structure and loosen the superhelix, resulting in the pathologies seen. The clinical, radiographic, and morphological features of the proband and affected members of the family in our report were consistent with the diagnosis of non-lethal type II collagen disorder, and significant overlap in phenotype was noticed among the patients. The molecular mechanisms of different type II collagenopathies may be driven not only by structural changes in the architecture of the extracellular collagenous matrices but also by intracellular processes activated by the presence of mutant collagen II molecules [18].

Analysis of exons 6–48 in the triple-helical domain of type II collagen has indicated that mutations in this region may have a dramatic effect on the function of the fibrillar collagen molecule. As a result of the relatively large size and complexity of the *COL2A1* gene and the variability of the mutant type, type II collagen is mainly expressed in cartilage instead of in cells. In the endoplasmic reticulum (ER) and Golgi of the chondrocyte, the pro-α1 chains form a triple helix and are secreted into the extracellular matrix (ECM) where they integrate and form an abundant collagen meshwork [19], [20], [21]. It has been reported that abnormal α1 chains, lacking several residues of the triple helical domain, are prominently incorporated into the structural collagen of cartilage in patients with collagenopathies. Given that the deleted sequences maintain the Gly–X–Y triple-helical motif, it is reasonable that molecules incorporating shorter α-chains can assemble into fibrils and become cross-linked, but that the normal distribution of intermolecular cross-linking cannot occur [22].

*Mortier et al.* [23] found that the family members with a glycine substitution mutation possessed significant amounts of post-translationally overmodified type II collagen in cartilage. The mutation in this domain hindered the progress of protein folding into the triple helical domain, which caused post-translational overmodification of the protein amino-terminal to the mutation site [24]. This is consistent with previous findings that an abnormal α1 chain with mutation in triple helical domain can be secreted into the cartilage. It has been reported that abnormal α1 chain may be expressed and incorporated into the matrix when a mutation has occurred in the donor splice site of the intron [23]. We believe that the abnormal α1 chain with the mutation found in this study (c. 1888 G>A) can be secreted and incorporated into the cartilage, resulting in a relatively mild phenotype, but the mechanism requires further investigation.

In our study, the major clinical manifestation in the proband and affected members of the family is in the hip joint, resulting in two different diseases, which differs from previous reports. Although both LCPD and ANFH are both collagenopathies, relevant studies have not reported the two diseases in one family with the same mutation. It has been suggested that an abnormality in type II collagen is an important factor in the development of both diseases, and they may represent multiple phenotypes resulting from a single underlying mechanism present with a family. The family member (IV-9) who carries the mutation without abnormality is advised that she may be in the presymptomatic stage. It has been shown that the femoral head epiphysis closes at 17–19 years in boys and at 15–17 years in girls in the Chinese population. LCPD emerges prior to the closure of the femoral head epiphysis, and ANFH emerges during the closure [6]. This suggested that the age at onset has an influence on the different phenotypes shown with the same mutation of the *COL2A1* gene. Many of the conditions present in childhood, but milder phenotypes presenting in adulthood are increasingly recognized [25]. The age of onset of the condition in most family members before 10-years (Table 1), however the age at onset in the condition in most patients with LCPD was earlier than the patients with ANFH. The X-ray of the family members III-6, III-7, IV-7, IV-8, and IV-9 were made at the age of 41, 38, 15, 15 and 7 years old respectively. In the present study, the family member (IV-9), containing the mutation in *COL2A1*, had no apparent change in her radiograph of the hip, and she should be followed up. In this situation, the mutation screening of this mutation proved to be an important diagnostic tool, and any preventive treatment can be made to the presymptomatic person (IV-9) in order to alleviate the severity of the disease when she grow up. The mechanism underlying the genotype-phenotype relationship needs further investigation based on the different mutations found in *COL2A1*.

In summary, our studies have discovered a novel substitution mutation (c. 1888 G>A) in the *COL2A1* gene. This mutation extends the mutation spectrum associated with collagenopathies and may be helpful in early molecular diagnosis. The underlying mechanism of mutation in the *COL2A1* gene that result in related diseases phenotypes requires further intensive studies.
